# An enzymatic system for decolorization of wastewater dyes using immobilized CueO laccase‐like multicopper oxidase on poly‐3‐hydroxybutyrate

**DOI:** 10.1111/1751-7915.13287

**Published:** 2018-06-12

**Authors:** Daniel Bello‐Gil, Emma Roig‐Molina, Jennifer Fonseca, María Dolores Sarmiento‐Ferrández, Marcela Ferrándiz, Esther Franco, Elena Mira, Beatriz Maestro, Jesús M. Sanz

**Affiliations:** ^1^ Instituto de Biología Molecular y Celular Universidad Miguel Hernández Avda. Universidad s/n 03202 Elche Spain; ^2^ Biotechnology Research Group Textile Research Institute (AITEX) Plaza Emilio Sala 1 03801 Alcoy Spain; ^3^ Centro de Investigaciones Biológicas Consejo Superior de Investigaciones Científicas c/Ramiro de Maeztu 9 28040 Madrid Spain; ^4^ Present address: Institut d'Investigació Biomédica de Bellvitge (IDIBELL) Barcelona Spain; ^5^ Present address: Centro de Investigaciones Biológicas (CSIC) Madrid Spain

## Abstract

The presence of synthetic dyes in wastewaters generated by the textile industry constitutes a serious environmental and health problem that urges the scientific community on an appropriate action. As a proof‐of‐concept, we have developed a novel approach to design enzymatic bioreactors with the ability to decolorize dye solutions through the immobilization of the bacterial CueO laccase‐like multicopper oxidase from *Escherichia coli* on polyhydroxybutyrate (PHB) beads by making use of the BioF affinity tag. The decolorization efficiency of the system was characterized by a series of parameters, namely maximum enzyme adsorption capacity, pH profile, kinetic constants, substrate range, temperature and bioreactor recycling. Depending on the tested dye, immobilization increased the catalytic activity of CueO by up to 40‐fold with respect to the soluble enzyme, reaching decolorization efficiencies of 45–90%. Our results indicate that oxidase bioreactors based on polyhydroxyalkanoates are a promising alternative for the treatment of coloured industrial wastewaters.

## Introduction

The presence of synthetic dyes in wastewaters generated by the textile industry constitutes a serious environmental problem that contributes to estimate this human activity as one of the most polluting worldwide (Sarayu and Sandhya, 2012; Verma *et al*., 2012; Zaharia and Suteu, 2012). Besides the toxic, carcinogenic, mutagenic and teratogenic properties of these substances, their accumulation also leads to highly opaque watercourses that prevents an adequate light entrance for the photosynthetic organisms to carry out their function normally, therefore resulting in poor oxygenation of the environment. It has been estimated that 2%–20% of the total annual production of textile dyes that are used in industry (about 7 × 10^5^ metric tonnes) are directly discharged as aqueous effluents to different watercourses (Zaharia and Suteu, 2012). Besides these environmental problems, textile industry also makes use of a large amount of water that should be recycled to the largest extent: on average, the daily water consumption of a medium‐sized company producing about 8000 kg of fabric per day is approximately 1.6 million litres (Rita, 2012).

Among the possible strategies to eliminate or reduce the presence of dyes in wastewaters, the use of enzymes with a broad substrate range has demonstrated to constitute one of the most promising methods. Thanks to their catalytic efficiency and their natural origin, these molecules are very suitable to be implemented in environmentally related processes such as pollutant biodegradation (Ahuja *et al*., 2004). In this sense, laccases (benzenediol: oxygen oxidoreductases; EC 1.10.3.2) have demonstrated an unquestionable biotechnological potential. These versatile, copper‐dependent enzymes belong to the super family of multicopper oxidases (Messerschmidt and Huber, 1990) and catalyse the oxidation of a wide variety of organic compounds including phenols, polyphenols, anilines and several inorganic compounds, either in soluble form or immobilized on solid supports (Fernandez‐Fernandez *et al*., 2013; Mate and Alcalde, 2016; Kudanga *et al*., 2017). The employment of laccases in dye decolorization has been widely described at the laboratory scale (Wesenberg *et al*., 2003; Rodriguez Couto and Toca Herrera, 2006; Giardina *et al*., 2010; Majeau *et al*., 2010). On the other hand, prokaryotic ‘laccases’ (more correctly referred to as laccase‐like multicopper oxidases) (Grass and Rensing, 2001; Reiss *et al*., 2013) have received relatively little attention compared to their fungal counterparts, yet they are especially attractive due to their broad substrate range and their ability to be cloned and overexpressed in *Escherichia coli* allowing their easy purification and immobilization with the eventual help of an affinity tag. Moreover, prokaryotic oxidases of this kind are usually more thermostable and halotolerant, operate in a wider pH range than the enzymes of fungal origin and are easily amenable to protein engineering manipulation (Singh *et al*., 2004; Brissos *et al*., 2009; Santhanam *et al*., 2011; Wang *et al*., 2012; Ihssen *et al*., 2015; Martins *et al*., 2015).

On the other hand, the use of enzymes at the industrial level is limited by their relatively reduced stability as well as the high costs and invested time related to their purification. A solution to these drawbacks is the immobilization of enzymes on a solid support, allowing their recycling and the precise control of catalysed reactions, as well as enhancing protein stability (Brena *et al*., 2013; Singh *et al*., 2013). Nevertheless, usual procedures of enzyme immobilization by adsorption such as immobilized metal affinity chromatography (IMAC) often make use of expensive matrices (Hochuli *et al*., 1987) or, in the case of covalent attachment, toxic reagents are needed to activate the enzyme or the support (Rusmini *et al*., 2007) and the structure of the protein may be severely affected (Mateo *et al*., 2007). Furthermore, gentler immobilization procedures (entrapment or encapsulation) are frequently associated with mass transfer limitations (Fernandez‐Fernandez *et al*., 2013). These aspects complicate the application of enzymatic systems in high‐scale procedures such as wastewater decolorization. In this sense, polyhydroxyalkanoates (PHAs) emerge as apt materials for many industrial and biomedical processes (Chen, 2009, 2011; Dinjaski and Prieto, 2015; Ke *et al*., 2017). Natural PHAs are organic polyoxoesters composed of (R)‐3‐hydroxy fatty acids which are synthesized in the cytoplasm of certain bacterial species and that accumulate as intracellular granules that constitute the carbon source and energy storage under nutrient limitation conditions (Madison and Huisman, 1999; Lenz and Marchessault, 2005). These polymers from natural sources show thermoplastic properties (hence the commonly used term ‘bioplastics’) and are considered environmentally friendly materials due to their biodegradability and biocompatibility properties, making them an attractive alternative to oil‐derived plastics in some applications (Chanprateep, 2010; Keshavarz and Roy, 2010; Park *et al*., 2012). As a consequence, PHAs have also attracted much attention to generate bioactive materials through the immobilization of proteins on their surface, leading to a number of potential biomedical applications (Brigham and Sinskey, 2012; Dinjaski and Prieto, 2015). However, the role of protein‐functionalized PHAs in other biotechnological fields such as those dealing with environmental issues (Moldes *et al*., 2006) deserves further exploration.

Based on this background, we have designed a system for dye decolorization using laccases or laccase‐like oxidases immobilized on PHAs, using the BioF tag. We have chosen polyhydroxybutyrate (PHB) as support, since it is the most commercially successful PHA and is present in a variety of manufactured products thanks to its versatility and cost‐effectiveness (Yu, 2014). Moreover, PHB has been the subject of several protein immobilization studies by a variety of procedures (Backstrom *et al*., 2007; Deepak *et al*., 2009; Yang *et al*., 2015; Beran *et al*., 2016; Rehm *et al*., 2017). Decolorization was tested with the broad substrate range, bacterial laccase‐like multicopper oxidase CueO, an enzyme that is involved in the copper homeostasis in *E. coli* (Grass and Rensing, 2001; Singh *et al*., 2004). Immobilization of CueO oxidase on PHB was achieved as a chimeric protein fused to the BioF affinity tag, the PHA binding domain of the PhaF phasin from *Pseudomonas putida* KT2442 (Prieto *et al*., 1999; Moldes *et al*., 2004, 2006). Although very interesting *in vivo* procedures have been described using fusions with the PhaC synthase from *Ralstonia eutropha* leading to covalent attachment to PHA granules *in vivo* (Rehm *et al*., 2016), we have recently demonstrated the feasibility of the non‐covalent interaction between PHB particles and the BioF tag for the construction of protein‐based biomaterials that demand, as in the present case, a good control of the amount of immobilized protein, versatility of available supports and easy matrix regeneration once the protein is inactivated (Bello‐Gil *et al*., 2018). Here, the degradation capacity of PHB minibioreactors containing the CueO enzyme was evaluated on different industrial dyes and in the absence of usual laccase mediators (Bourbonnais *et al*., 1997; Moldes and Sanroman, 2006; Ibarra *et al*., 2007) with a view on decreasing process costs upon future scaleup. Our results demonstrate a new utility of PHAs as biomaterials for the enzymatic treatment of textile industry effluents upon functionalization with laccase‐like oxidases.

## Results and Discussion

### Enzymatic parameters of the hybrid BioF–CueO protein

Although many oxidases from bacterial or fungal origin might possess better biotechnological properties in certain cases (Wesenberg *et al*., 2003; Rodriguez Couto and Toca Herrera, 2006; Giardina *et al*., 2010; Majeau *et al*., 2010), we chose the CueO multicopper oxidase to establish the present proof‐of‐concept since this enzyme has already been thoroughly characterized both structurally and functionally for the oxidation of several organic substrates, including dyes (Zeng *et al*., 2011; Britos and Trelles, 2016; Ma *et al*., 2017)

To ensure a strong and specific adsorption of the CueO oxidase to PHB, a chimeric protein was constructed containing the BioF tag fused to the N‐terminal of the CueO enzyme (Fig. S1). The BioF–CueO protein was overexpressed and purified as described in Experimental Procedures (Fig. S3). The pH profile in solution of the hybrid BioF–CueO for 2,6‐dimethoxyphenol (DMP) oxidation displays a maximum at pH 6.5 (Fig. S4), as previously described for non‐fused CueO (Roberts *et al*., 2002). Moreover, the Michaelis–Menten analysis of the oxidase activity on DMP at pH 6.5 and 30 °C (Fig. S5) yielded kinetic constants that are shown in Table 1. The kinetic parameters were obtained in a low Cu^2+^ concentration (10 μM) to diminish nonspecific oxidation of DMP caused by the metal, and that kinetic parameters are highly dependent on copper concentration (Kim *et al*., 2001; Roberts *et al*., 2002; Kataoka *et al*., 2007; Djoko *et al*., 2010).

**Table 1:** Kinetic parameters for DMP oxidation by soluble and immobilized BioF–CueO^a^

Enzyme	*K*_M_ (mM)	*k*_cat_ (s^−1^)	*k*_cat_/*K*_M_ (s^−1^ mM^−1^)
Soluble	0.4 ± 0.1	4.3 ± 0.1	10.7
Immobilized	0.8 ± 0.1	1.7 ± 0.1	2.1

a Mean of duplicates plus standard deviation.

To determine the activity of the immobilized BioF–CueO, adsorption of the enzyme on commercial PHB particles was achieved by direct mixing of the protein solution with the matrix. Langmuir analysis of the binding isotherm at 25°C (Eq. (1), Fig. S6) yielded a dissociation constant of 0.7 ± 0.1 mg ml^−1^ and a maximum adsorption of 40 ± 2 μg of protein per mg of support. The affinity is similar to that displayed by the PhaF phasin in this system (*K*_d_ = 0.5 mg ml^−1^) although the binding capacity for BioF–CueO is appreciably higher (only 20 μg of protein per mg of support for PhaF) (Bello‐Gil *et al*., 2018). Calculation of these features was essential to design the enzymatic experiments using immobilized protein, since they indicate which should be the initial soluble protein concentration to incubate with PHB for a given defined amount of actual immobilized enzyme per mg of support.

The kinetic analysis of the activity of immobilized BioF–CueO on DMP shows that adsorption causes a moderate increase of the apparent *K*_M_ (Table 1) which may be ascribed to a restricted substrate diffusion to the enzymatic phase in the presence of the macroscopic support. Furthermore, a decrease in *k*_cat_ was also observed compared to the soluble protein, which might be taken in the context of a necessarily indirect estimation of total protein bound to the support using Eq. (1) (see above). On the other hand, the pH–activity profile of the immobilized protein is undistinguishable from the soluble form (Fig. S4). Moreover, due to the mentioned technical limitations with DMP, the dependence of oxidase activity on Cu^2+^ concentration was analysed instead by measuring the oxidation of the Reactive Black 5 (RB5) dye (Fig. S7), as in this case the nonspecific effect of the metal was found to be negligible (data not shown). The Michaelis constant of BioF–CueO for Cu^2+^ was calculated as *K*_M_ = 0.15 ± 0.05 mM, close to the value of 0.16 mM reported before for CueO on DMP (Roberts *et al*., 2003) and therefore suggestive of being mostly independent of the substrate used. All these data suggest that the conformation of the CueO moiety is not appreciably affected by binding to the PHB granules.

### Substrate range of immobilized BioF–CueO

**Figure 1:**
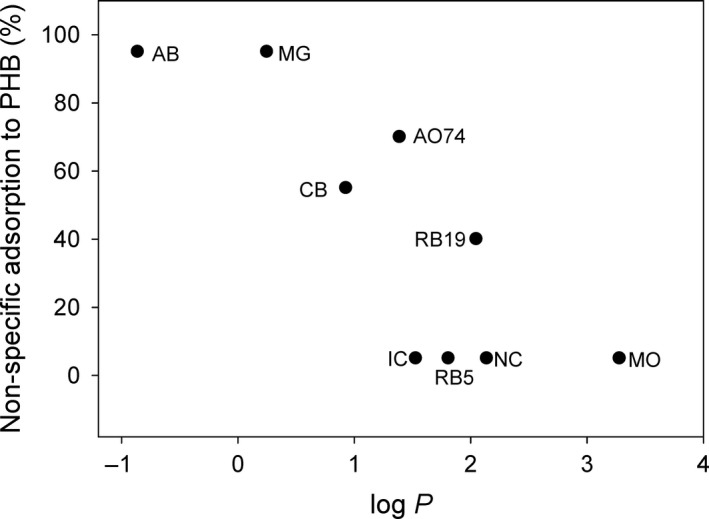
Correspondence between the estimated log*P* for each dye and their nonspecific adsorption to PHB.

A few studies have previously analysed the scope of substrates able to be oxidized by CueO (Kataoka *et al*., 2007; Li *et al*., 2007; Zeng *et al*., 2011), but have only recently included dye molecules (Britos and Trelles, 2016; Ma *et al*., 2017). Therefore, we aimed to study the activity of PHB‐immobilized CueO on some representative dyes that were chosen to cover different structural families (Fig. S2 and Table 2). We set our standard decolorization conditions to pH 7.0 since laccases present very different optimal pH values for their activity depending on each particular substrate, but incubation at acidic pH for long times may result in some protein denaturation (Kunamneni *et al*., 2007). Two different behaviours could be discerned: (i) Dyes which totally or partially interacted with PHB, namely: Cibacron Blue 3GA (CB), Reactive Blue 19 (RB19), Acid Orange 74 (AO74), Azure B (AB) and Malachite Green (MG); and (ii) dyes showing no appreciable adsorption on PHB, namely: Methyl Orange (MO), New Coccine (NC), Reactive Black 5 (RB5) and Indigo Carmine (IC). Only a few studies have reported direct interactions between PHB and dyes (Sudesh *et al*., 2011), including the strong MG–PHB interaction (Sridewi *et al*., 2011). It is noteworthy that there is an apparent correlation between the degree of interaction with PHB and the polarity of each dye (represented by its estimated log*P* coefficient, see Experimental Procedures), so that the most polar compounds seem to be preferentially adsorbed to the polymer (Fig. 1, Table 2). This observation deserves to be further investigated in order to check the feasibility of using non‐functionalized polyhydroxyalkanoates to decolorize wastewaters containing certain dyes. In any case, since the aim of our work was to focus on CueO‐mediated dye degradation, those compounds that showed the highest binding to PHB (that is AO74, AB and MG) were discarded for our studies.

**Table 2:** Dyes analysed in this study

Dye	Type	Wavelength (nm)	E^0.1%^ (ml mg^−1^ cm^−1^)^a^	Estimated log*P*	Adsorption to PHB (%)^b^
Azure B (AB)	Heterocyclic	647	130.0	−0.9	>95
Malachite Green (MG)	Triarylmethane	615	666.7	0.3	>95
Cibacron Blue 3GA (CB)	Anthraquinone	612	14.4	0.9	55 ± 6
Reactive Blue 19 (RB19)	Anthraquinone	592	9.3	2.1	40 ± 1
Acid Orange 74 (AO74)	Azo	455	17.9	1.4	70 ± 5
Methyl Orange (MO)	Azo	465	71.4	3.3	<5
New Coccine (NC)	Azo	506	41.7	2.1	<5
Reactive Black 5 (RB5)	Azo	597	25.0	1.8	<5
Indigo Carmine (IC)	Indigoid	608	40.3	1.5	<5

a Determined in 20 mM sodium phosphate buffer pH 7.0 plus 1 mM Cu^2+^.

b Twenty millilitres of each dye solution (1 absorbance unit) in 20 mM sodium phosphate buffer pH 7.0 plus 1 mM Cu^2+^ were incubated with PHB (50 mg mL^−1^) in a shaker incubator at 150 rpm and 25°C for 90 h. Samples were then centrifuged at 10 000× *g* for 10 m and the absorbance of the supernatant was measured.

### BioF–CueO activity on partially adsorbed dyes

**Figure 2:**
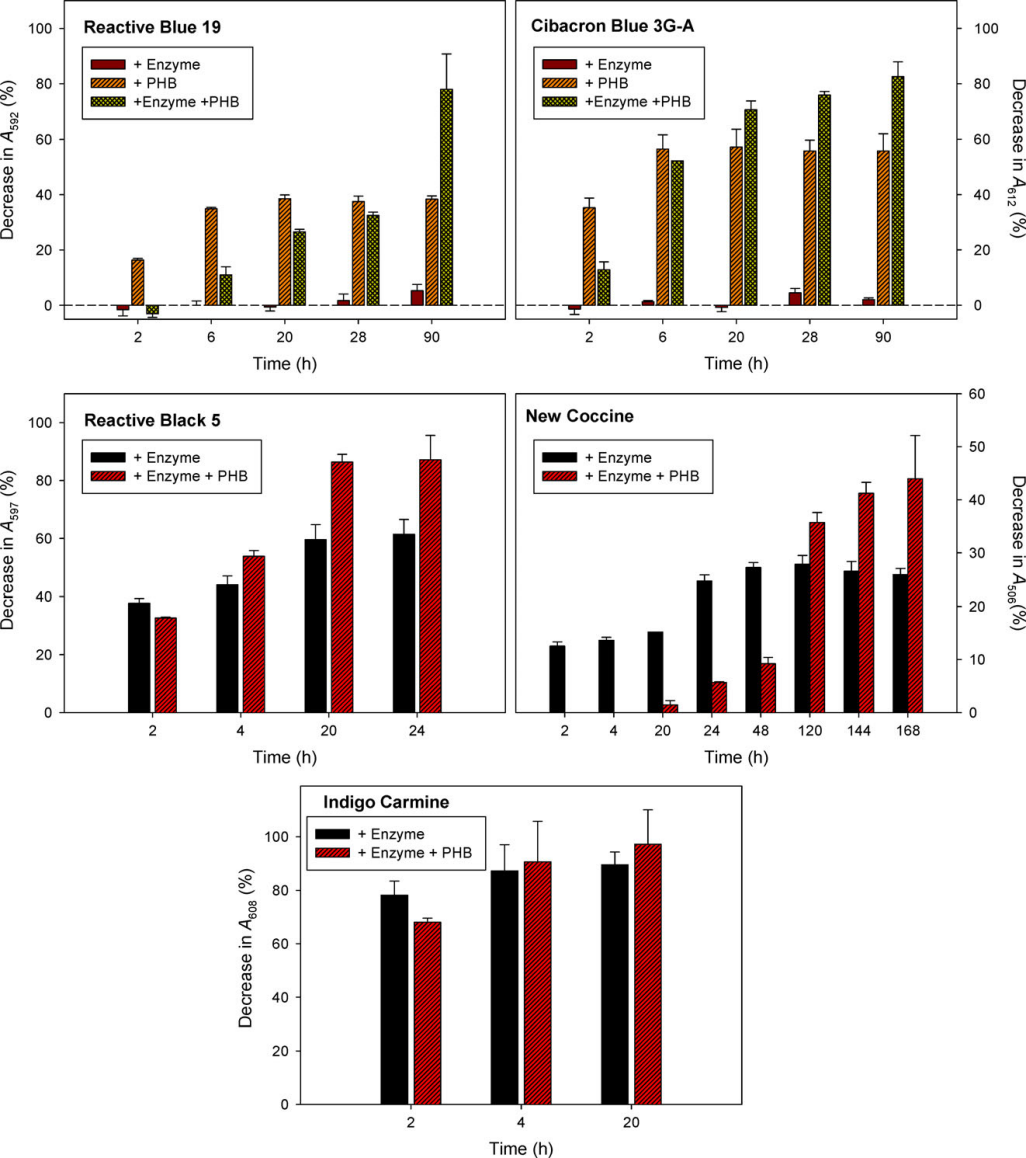
Decolorization of textile dyes by BioF–CueO. Samples (20 mL) were incubated in batch with PHB‐immobilized BioF–CueO by shaking at 150 r.p.m. A blank containing no enzyme or support was subtracted in all cases. Results are the mean of triplicate experiments and are shown as decrease in absorbance with respect to the initial values. The first two plots (Reactive Blue 19 and Cibacron Blue 3G‐A) correspond to dyes that partially adsorb to PHB, while the last three plots (Reactive Black 5, New Coccine and Indigo Carmine) correspond to dyes showing negligible adsorption (<5%) to PHB.

**Figure 3:**
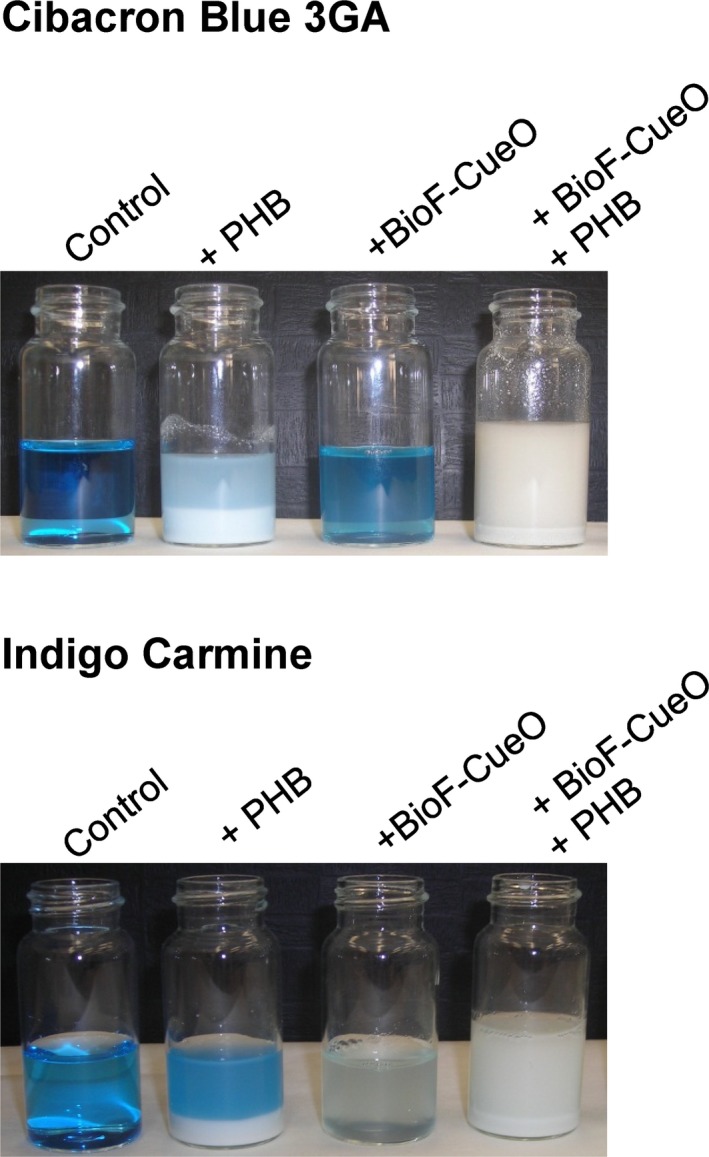
Examples of dye degradation with BioF–CueO after 20 h incubation. Conditions as detailed in Fig. 2.

CB and RB19, both anthraquinone dyes, were partially adsorbed to PHB in a time‐dependent fashion, reaching maximum binding after a 6 h of incubation (Fig. 2). On the other hand, they demonstrated to be very poor substrates for soluble BioF–CueO even after 90 h of incubation (Figs 2 and 3). Strikingly, when the enzyme was bound to the support, a decrease in dye removal was detected in the short time scale compared to the support alone. This phenomenon might be explained in terms of a relevant surface of the granule being coated with protein, therefore sterically impeding the access of the dye. Nevertheless, longer incubation times finally led to a high removal efficiency (around 80%) corresponding to 1.5 (CB) and 2.1 (RB19) times than that of PHB alone (Figs 2 and 3). Moreover, compared to the soluble enzyme, this approximately represents. a 40‐ (CB) and 15‐fold (RB19) increase in catalytic activity after 90 h of incubation. (Fig. 2) Such an important decolorization increment may be due to an increase in the apparent affinity for the dyes caused by the preferential accumulation of these substrates in the surface of the PHB granule, a common support‐dependent effect described in general protein immobilization procedures (Dwevedi, 2016).

### BioF–CueO activity on non‐adsorbed dyes

Contrary to the previous case, those dyes with low nonspecific binding to PHB turned out to be good substrates for soluble BioF–CueO, which, at 25 °C and pH 7.0 reached its maximum decolorizing activity after c.a. 24 h of incubation (Figs 2 and 3). On the other hand, the immobilization of the recombinant laccase‐like protein led to a decreased activity in the short term (probably arising from a higher apparent *K*_M_ as discussed above – see Table 1 and Fig. S5), while longer incubation times allowed appreciable decolorizing efficiencies (reaching almost 100% for IC, Figs 2 and 3), resulting in improvements from 1.1‐fold (IC) to 1.4‐fold (RB5) and 1.7‐fold (NC) compared to the soluble protein. In particular, immobilized CueO‐mediated degradation of IC at neutral pH reached levels around 90% after 4 h, appreciably higher than those previously reported at pH 4.5 (7%) (Britos and Trelles, 2016). A striking case was methyl orange (MO), which was not degraded at all either by the soluble or immobilized enzyme in the tested conditions (data not shown). This is in accordance with the low activity reported by Pardo *et al*. on this substrate in the absence of mediators, that has been explained in terms of the lack of hydroxyl substituents in the molecule that would help promoting oxidation, leading to a very high redox potential (Pardo *et al*., 2013). Nevertheless, a somewhat higher degradation of MO has been reported to occur at more acidic pH values (Britos and Trelles, 2016).

Data shown in Fig. 2 indicate that, at 25°C, the BioF–CueO enzyme in solution achieves maximal levels of activity after approximately 24 h of incubation, while immobilization on the PHB particles extends the performance of the enzyme even up to 168 h (NC).

### Optimization of BioF–CueO performance

Once set the characteristics of BioF–CueO in terms of kinetic constants, immobilization on PHB and substrate range, we decided to optimize the functioning conditions on a laboratory‐scale enzymatic minibioreactor to establish a proof‐of‐concept for the use of this system in a larger scale. With this aim, our efforts were dedicated to reduce the amount of Cu^2+^ needed, as well as to evaluate the recycling and reuse the minibioreactor.

**Figure 4:**
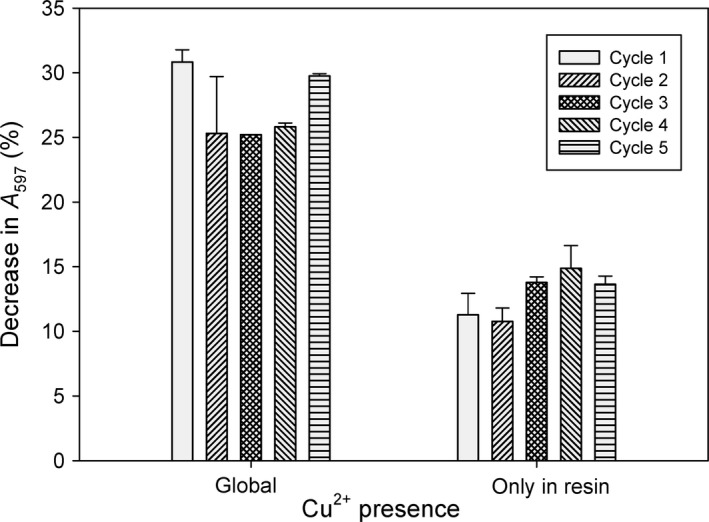
Optimization of Cu^2+^ addition. An RB5 solution (1 mL) was incubated in batch on a digital rotary mixer for 1 h with PHB (approximately 0.1 mL of bed volume) functionalized with BioF–CueO as described in Experimental Procedures. After each incubation cycle, the supernatant was removed and analysed, and the resin was washed with Tris buffer and then loaded with a fresh dye solution for a subsequent decolorization cycle. *Global* means that 0.1 mM Cu^2+^ was maintained constant in the dye solution during the decolorization process. *Only in resin* means equilibration of the PHB support with 0.1 mM Cu^2+^ (0.1 mL), followed by washing and subsequent addition of dye solution without the metal ion. Results (mean of triplicates) are shown as decrease in absorbance with respect to the initial values.

Due to the presence of a labile copper binding site, the CueO enzyme is highly dependent on the presence of Cu^2+^ in the medium to develop its activity (Roberts *et al*., 2003) (Fig. S7). However, addition of high concentrations of this metal to the considerable volumes of industrial wastewaters would pose negative effects on the environment. Therefore, we decided to investigate an alternative procedure to reduce as much as possible the total amount of Cu^2+^ needed for dye decolorization. In this sense, since the BioF–CueO oxidase is immobilized in a small volume of PHB compared to the solution to be treated (approximately 1:10 v/v), we determined the possibility of employing in first place only the amount of Cu^2+^ to saturate the immobilized protein on the resin, and then assaying the dye solution free of metal. Fig. 4 (left) shows that, in the overall presence of 0.1 mM Cu^2+^, an RB5 solution could be decolorized to a substantial 30% and the support could be reused in these conditions at least five times without appreciable loss of activity. On the other hand, adding only the necessary metal to saturate the resin‐bound protein previously to the assay, the enzymatic activity decreased two‐fold (Fig. 4, right). Nevertheless, the use of Cu^2+^ was reduced in a 90% compared to the first situation, and the reusability was also unaffected. These results might be useful when considering the scaling up of the system, with the aim of finding the most appropriate balance between maximum enzymatic capacity and environmental side effects.

Figure 4 shows that BioF–CueO‐funcionalized PHB can be reused at least in the short term (five 1 h cycles). This is in accordance with the ability of a BioF–β‐galactosidase fusion adsorbed on PHB to maintain its enzymatic function after several activity + washing cycles (Bello‐Gil *et al*., 2018). To evaluate reusability at longer incubation times and with a different dye, we analysed the activity of the minibioreactor on IC after several 20 h of cycles. Results displayed in Supplementary Fig. S8 indicate that the enzyme is fully active after four rounds (amounting to 90 h of continuous use), and only after this time an appreciable inactivation was observed. This suggests that immobilized BioF–CueO enzyme may be reused several times in long cycles, allowing the degradation of dyes with slow enzymatic kinetics that require extensive reaction times.

Thanks to the non‐covalent interaction between BioF–CueO and PHB, once the enzyme is inactivated, the support can be easily regenerated by the simple addition of surfactants that cause protein elution (Moldes *et al*., 2004). Figure S9 shows that addition of sodium dodecyl sulphate (SDS) and subsequent washing rendered a PHB matrix able to bind fresh recombinant enzyme with the same efficiency.

### Effect of temperature

**Figure 5:**
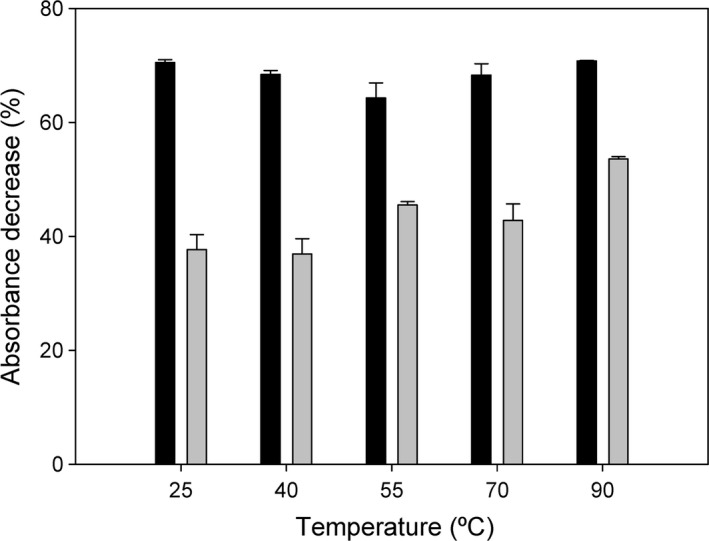
Effect of temperature on BioF–CueO‐mediated dye degradation. Solutions of RB5 (black bars) or IC (grey bars) in 20 mM Tris buffer, pH 7.0, plus 0.1 mM CuSO_4_ were preincubated for 30 min at the temperatures shown in the graphs, then added to the functionalized resin and left to react at 25 °C for 24 h. Blanks (enzyme‐free) were subtracted in each experiment. Results (mean of triplicates) are shown as decrease in absorbance with respect to the initial values.

Although all characterization experiments shown above were carried out at moderate temperatures (25–30°C), it should be taken into account that the majority of textile industry wastewaters abandon the dyeing baths at high temperatures, and that should refrigeration be necessary, this would add costs and time to the degradation procedure. In this sense, and based on the previously described thermal stability shown by CueO (maximum activity at 55°C) (Roberts *et al*., 2002; Ma *et al*., 2017), we determined the possibility of using the BioF–CueO biorreactor to decolorize solutions initially preincubated at different temperatures, so that dye degradation occurs simultaneously with spontaneous cooling down to room temperature. Results shown in Fig. 5 demonstrate that, at least in our small‐scale system, final enzymatic activity is not affected despite being challenged with dye solutions initially heated up to 90°C. This suggests that wastewaters can be subjected directly to enzymatic degradation by PHB‐immobilized BioF–CueO after the dyeing process without any prior refrigeration.

### Assay on industrial wastewaters

**Figure 6:**
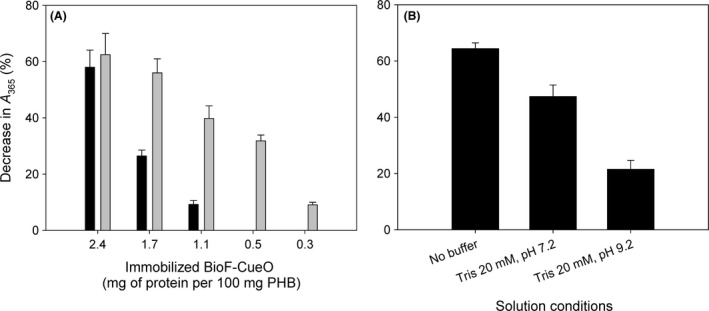
*A*, Decolorization of an industrial wastewater sample by immobilized BioF–CueO in the absence (black bars) or the presence (grey bars) of 0.1 mM Cu^2+^. *B*, Effect of pH and solution conditions on activity. Samples contained 1.7 mg of protein per 100 mg of PHB.

Enzymes are usually prone to inactivation by many compounds that may appear in common wastewaters from the textile industry. With the aim of emulating such conditions, we first assayed the PHB/CueO minibioreactor on RB5 solutions containing usual additives employed in dyeing procedures such as salts (Na_2_SO_4_, 20 mg ml^−1^), tensoactives (Auxigal PRD, 2 μl ml^−1^) or dispersants (CI‐HT, 2 μl ml^−1^). None of these compounds affected the decolorizing activity to an appreciable extent (Fig. S10). This prompted us to analyse the activity of the enzymatic bioreactor on a sample of industrial wastewater sample supplied by the Textile Research Technological Institute (AITEX, Alcoy, Spain) that contained a mixture of three dyes, namely Direct Red 105, Direct Yellow 106 and Direct Black 112 (see Materials and Methods). The pH of the effluent sample was 6.7–7.1, depending on the aliquot. No change in absorbance was detected upon incubation of the sample with PHB alone, ruling out any nonspecific adsorption of the dyes (data not shown). As depicted in Fig. 6, the decolorizing capacity of immobilized BioF–CueO already demonstrated for standard dye solutions is reproduced in the case of a real industrial effluent. Moreover, while addition of Cu^2+^ clearly enhanced the degradation activity independently of the enzymatic load, these differences were drastically reduced when the resin approached protein saturation (Fig. 6A). On the other hand, Fig. 6B shows an appreciably decrease in activity in Tris buffer at alkaline pH (9.2) compared to neutral (pH 7.2), in line with the pH profile shown in Fig. S4. Remarkably, the decolorization activity on non‐buffered sample increased in c.a. 20% compared to the Tris‐buffered sample at the same pH (Fig. 6B). This might be explained by the Cu^2+^ chelating effect displayed by the Tris molecule that has been described to negatively affect CueO activity (Djoko *et al*., 2010) and suggests that pH adjusting might not be needed for wastewaters provided their pH is suitable for CueO activity (Fig. S4), therefore allowing a substantial reduction in industrial costs. These results altogether confirm the reliability of the PHB/oxidase system and reinforce its potential to be employed at industrial scale.

## Conclusions

We provide a proof‐of‐concept to evidence the use of enzymes immobilized on PHB bioplastics to decolorize solutions with industrial dyes using environmentally friendly supports such as polyhydroxyalkanoates. The system is efficient, simple, fully reusable and recyclable, provides stability to the immobilized enzyme and is adaptable to environmental conditions such as high temperatures and the presence of usual textile additives in the solution. The versatility of the BioF system foresees an easy and strong binding of other laccase or laccase‐like oxidases to PHAs while maintaining or even expanding their activity (Fig. 2). This use of PHAs as supports for enzymatic dye degradation, constitutes therefore a significant added value for these biopolymers to help boost their industrial application. Future efforts might be aimed towards the *in vivo* simultaneous expression and binding of BioF‐tagged oxidases to PHA granules that can be easily recovered from bacteria (Moldes *et al*., 2006) (Moldes *et al*., 2004), thus making protein purification unnecessary and therefore decreasing downstream processing costs.

## Experimental procedures

### Construction of recombinant vector for BioF–CueO overexpression

The general cloning scheme is shown in Supplementary Information (Fig. S1). Plasmid pNFA2, coding for the hybrid BioF–C‐LytA (FLyt) protein (Moldes *et al*., 2004), was subjected to PCR using the BioF_XbaI (5′‐CGCATAAAGCTCTAGAAATAATTTTGTTTAACTTTAAGAAGGAGAATACGATGGCTGGCAAGAAGAATTCCGAG‐3′) and BioF_NcoI (5′‐TCGTTCCGCTGA‐TCCATGGGCTGCTGCCCCGCGACGAAATCGGGGTAACC‐3′) primers (*Xba*I and *Nco*I sites are underlined). The amplified fragment was purified by the ‘High Pure PCR Product Purification’ kit (Roche Applied Science), digested with *Xba*I and *Nco*I (Fermentas) and cloned between the *Xba*I and *Nco*I sites of pET‐21d (+) vector (Novagen), yielding intermediate plasmid pDB1. Next, the *cueO* gene was amplified by PCR from plasmid pCueO, a derivative of pASK‐IBA3 (Grass and Rensing, 2001)} that was generously provided by Prof. William R. Montfort. Primers used were CueO_NcoI (5′‐ CGCATAAAGCCCATGGGCAGAACGCCCAACGTTACCGAT‐3′) and CueO_BamHI (5′GCTTTATGCGGGATCCTTATTATACCGTAAACCCTAACATCATCCC‐3′) (*Nco*I and *Bam*HI sites are underlined). The purified PCR fragment was then ligated as a *Nco*I/*Bam*HI DNA fragment into pDB1, yielding plasmid pDB2, that expresses the *bioF–cueO* hybrid gene under the control of the T7*lac* promoter. The fidelity of all constructions was checked by DNA sequencing (Secugen, Madrid).

### Expression and purification of BioF–CueO

*Escherichia coli* BL21 (DE3) competent cells (Studier and Moffatt, 1986; Studier *et al*., 1990) were transformed with plasmid pDB2 using the CaCl_2_ transformation protocols (Sambrook and Russell, 2001). An overnight culture of freshly transformed cells grown in Luria–Bertani (LB) medium (Sambrook and Russell, 2001) was diluted 1:100 with fresh medium containing ampicillin (0.1 mg ml^−1^) and incubated at 37°C with shaking (200 rpm) until the culture reached an O.D._600_ of 0.6. Overexpression of *bioF–cueO* gene was then induced for 16 h at 25°C by addition of 0.5 mM isopropyl β‐D‐thiogalactoside (IPTG). Cells were harvested at 4°C, resuspended in 20 mM Tris buffer pH 7.0 plus 1 mM CuSO_4_ (1/20th of the original culture volume) and disrupted by sonication (Branson 250 instrument). The lysate was centrifuged at 4°C (10 000× *g*, 10 min). Ammonium sulfate was then slowly added upon stirring to the clear extract up to a final 0.75 M concentration and centrifuged again at 4 °C (10 000× *g*, 10 min). The supernatant was loaded into a butyl sepharose column (GE Healthcare) (10 cm × 1 cm) equilibrated in the same buffer as the sample. Extensive washing was carried out with 20 mM Tris buffer pH 7.0 plus 0.5 M ammonium sulfate, and the BioF–CueO protein was finally eluted with 20 mM Tris buffer pH 7.0, plus 0.3 M ammonium sulfate. Sample purity was evaluated by SDS‐polyacrylamide gel electrophoresis (PAGE) (Laemmli, 1970). The protein concentration was evaluated by absorption spectroscopy using a molar absorption coefficient at 280 nm of 81 025 M^−1^ cm^−1^ calculated using the online software ProtParam from the Expasy toolbox (http://web.expasy.org/protparam). Enzyme fractions were stored at 4°C until further use.

### Standard activity assay of BioF–CueO oxidase

Kinetic parameters of BioF–CueO were determined by monitoring the oxidation of 2,6‐dimethoxyphenol (DMP) at 30°C, as described before (Grass and Rensing, 2001). Experiments (in duplicate) were carried out at 30°C in 20 mM Tris buffer, pH 6.5, plus 10 μM Cu^2+^ and using a protein concentration of 1 μg ml^−1^. Addition of higher Cu^2+^ concentrations induced an intense nonspecific oxidation of the substrate that interfered with the monitoring of the enzymatic activity. Activity was determined spectrophotometrically at 468 nm in an Evolution 201 spectrophotometer (Thermo Scientific) following the appearance of the DMP oxidation product, 3,3′,5,5′‐tetramethoxydiphenoquinone (ε_468_ = 14 800 M^−1^ cm^−1^) (Slomczynski *et al*., 1995). The kinetic parameters (*K*_m_ and *k*_cat_) of the enzyme were calculated with the Michaelis Menten equation (Michaelis and Menten, 1913) using the SigmaPlot 10.0 utilities (Systat Software Inc.).

All graphs presented in this work were created with SigmaPlot 10.0. Data represent the mean of duplicate or triplicate experiments, depending on each case, and error bars represent the standard deviation.

### Protein immobilization on PHB

The affinity of the BioF–CueO enzyme for PHB interaction and the maximum protein binding capacity were assessed by Langmuir analysis (Hung *et al*., 2008). Commercial PHB (Sigma‐Aldrich) was used in our study. A thorough biophysical analysis of this material has been recently reported (Bello‐Gil *et al*., 2018). Ten milligrams of PHB particles were first washed with 20 mM Tris buffer pH 7.0 and incubated with increasing concentrations of the protein (0.1–2 mg ml^−1^) in the same buffer (600 μl). After mild mixing (30 m at 25°C) on digital rotary mixer (OVAN), the unbound protein fraction was recovered from the polyester by centrifugation (10 000× *g*, 3 min) and quantified spectrophotometrically. The amount of BioF–CueO bound to PHB (μg of protein/mg PHB) was calculated as the difference between total added protein and unbound protein. Data (mean of duplicates) were fitted to Eq. (1):

(1) $$q=\frac{q_{\max}C}{K_{d}+C},$$

where *q* is the amount of protein bound on PHB (μg protein/mg PHB), *q*_max_ is the maximum adsorption capacity of PHB granules (μg protein/mg PHB), *C* is the concentration of added protein (mg ml^−1^) and *K*_d_ is the dissociation constant (mg ml^−1^).

### Decolorization assays

Dye decolorization by BioF–CueO was determined by monitoring the change in absorbance at the maximum wavelength (λ_max_) for each dye (Table 2). Assayed dyes were Cibacron Blue 3GA (CB), Reactive Blue 19 (RB19), Methyl Orange (MO), Acid Orange 74 (AO74), New Coccine (Acid Red 18) (NC), Reactive Black 5 (RB5), Azure B (AB), Indigo Carmine (Acid Blue 74) (IC) (Sigma‐Aldrich) and Malachite Green (MG) (Fischer Scientific) (Fig. S2). Decolorization is reported as:

(2) $$\text{Decolorization}\left(\%\right)=\frac{A_{i}-A_{f}}{A_{i}}\times 100,$$

where *A*_i_ is the initial absorbance and *A*_f_ is the final absorbance of the dye solution.

Unless otherwise stated, the standard decolorization assay was performed in triplicate at 25°C on a dye solution in 20 mM Tris buffer, pH 7.0, plus 1 mM Cu^2+^, initially set to 0.5 absorbance units. For immobilized protein, the solution was incubated with PHB (50 mg ml^−1^) containing 20 μg of adsorbed BioF–CueO per mg of polymer, in orbital shaker (200 rpm, INNOVATM 4000). Control experiments for soluble protein were carried out at the same final enzyme concentration but in the absence of PHB. For decolorization of real industrial wastewaters, a sample supplied by the Textile Research Technological Institute (AITEX, Alcoy, Spain) containing a mixture of Direct Red 105, Direct Yellow 106 and Direct Black 112, was diluted 1:40 (w/w) in 20 mM Tris buffer, pH 6.5, plus 0.1 mM Cu^2+^ (*A*_365_ = 0.2) and incubated with PHB (50 mg ml^−1^) containing variable amounts of adsorbed BioF–CueO (as estimated by Eq. (1)) in a total volume of 2 ml for 1 h at 25°C. Activity was measured by following the change in absorbance at 365 nm (the maximum of the wastewater sample absorbance spectrum).

Hydrophobicity of the dyes was estimated by calculating the logarithm of the n‐octanol/water partition coefficient (log*P*) with the ALOGPS 2.1 utility (http://www.vcclab.org/lab/alogps/) (Tetko *et al*., 2005).

## Conflict of interest

None declared.

## Supporting information


**Fig. S1.** Upper panel, Cloning scheme leading to plasmid pDB2 which overproduces the BioF‐CueO protein.
**Fig. S2.** Structures of dyes used in this work.
**Fig. S3**. SDS‐PAGE analysis of BioF‐CueO expression and purification.
**Fig. S4.** pH dependence of BioF‐CueO activity at 30°C both in aqueous solution and immobilized on PHB.
**Fig. S5**. Kinetic analysis of the activity of BioF‐CueO in solution (black) and immobilized on PHB (red).
**Fig. S6.** Langmuir isotherm of BioF‐CueO binding to PHB. Results are the mean of duplicates.
**Fig. S7.** Effect of Cu2+ concentration on the activity of BioF‐CueO immobilized on PHB.
**Fig. S8**. Batch reutilization of PHB‐immobilized BioF‐CueO for decolorisation of Indigo Carmine (IC) solutions.
**Fig. S9.** Recycling of bioactive PHB support.
**Fig. S10**. Decolorisation of a RB5 solution in the presence of additives.
