# Supplementary material for: An enzymatic system for decolorization of wastewater dyes using immobilized CueO laccase‐like multicopper oxidase on poly‐3‐hydroxybutyrate
Source: Microb Biotechnol. 2018 Jun 12;11(5):881–92. doi: 10.1111/1751-7915.13287 (PMC6116751; doi:10.1111/1751-7915.13287)
Supplement: Supplementary file 1 — Fig. S1. Upper panel, Cloning scheme leading to plasmid pDB2 which overproduces the BioF‐CueO protein. Fig. S2. Structures of dyes used in this work. Fig. S3. SDS‐PAGE analysis of BioF‐CueO expression and purification. Fig. S4. pH dependence of BioF‐CueO activity at 30°C both in aqueous solution and immobilized on PHB. Fig. S5. Kinetic analysis of the activity of BioF‐CueO in solution (black) and immobilized on PHB (red). Fig. S6. Langmuir isotherm of BioF‐CueO binding to PHB. Results are the mean of duplicates. Fig. S7. Effect of Cu2+ concentration on the activity of BioF‐CueO immobilized on PHB. Fig. S8. Batch reutilization of PHB‐immobilized BioF‐CueO for decolorisation of Indigo Carmine (IC) solutions. Fig. S9. Recycling of bioactive PHB support. Fig. S10. Decolorisation of a RB5 solution in the presence of additives. [file MBT2-11-881-s001.pdf]

An Enzymatic System for Decolorisation of Wastewater Dyes Using Immobilized CueO Laccase-like Multicopper Oxidase on Poly-3-hydroxybutyrate

Daniel Bello-Gil, Emma Roig-Molina, Jennifer Fonseca, M. Dolores Sarmiento-Ferrández, Marcela Ferrándiz, Esther Franco, Elena Mira, Beatriz Maestro, Jesús M. Sanz.

SUPPLEMENTARY INFORMATION

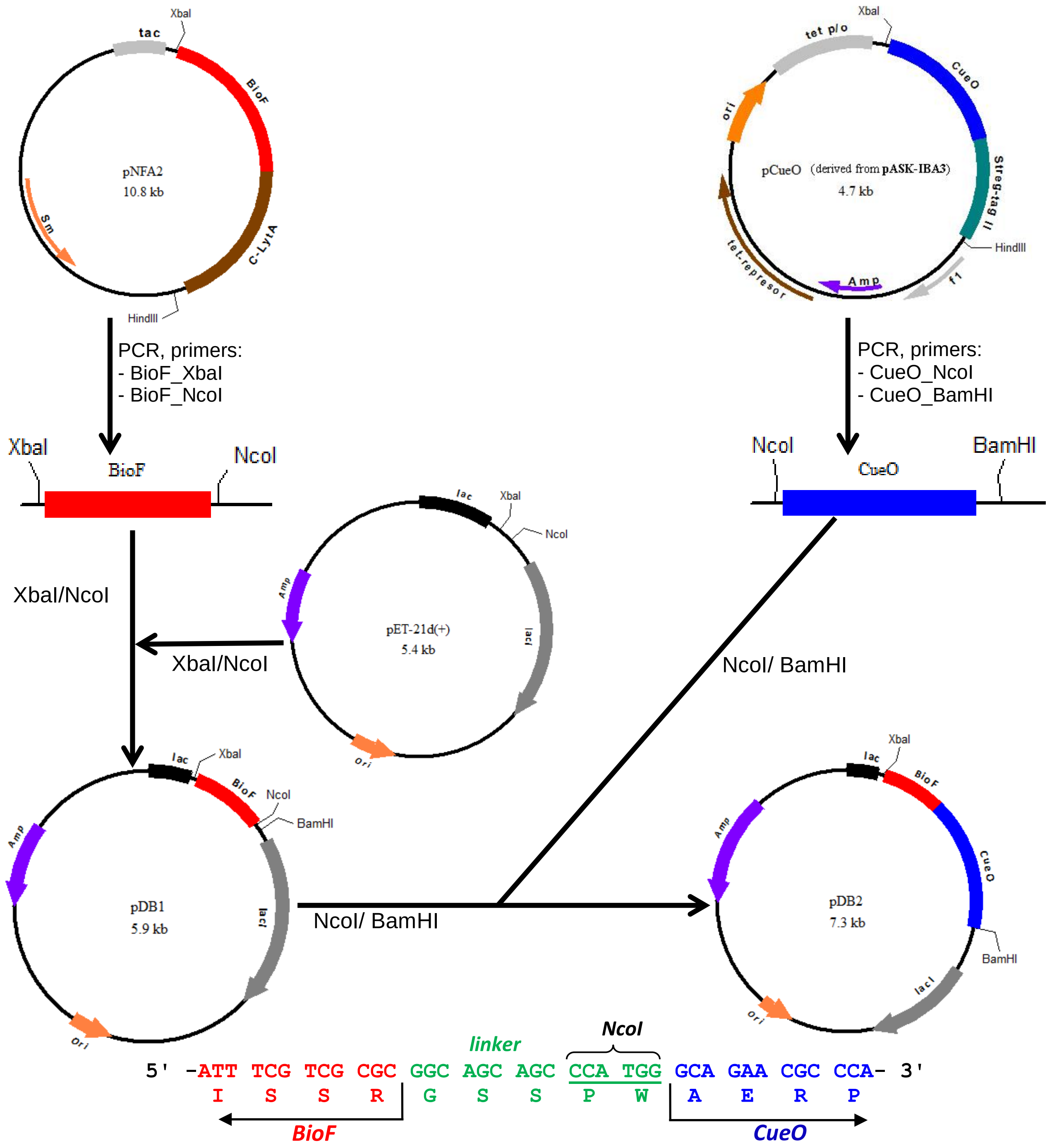

Fig. S1. Upper panel, Cloning scheme leading to plasmid pDB2 which overproduces the BioF-CueO protein. *Sm*, streptomycin resistance gene; *Amp*, ampicillin resistance gene. Lower panel: final sequence at the junction of *bioF* and *cueO* genes.

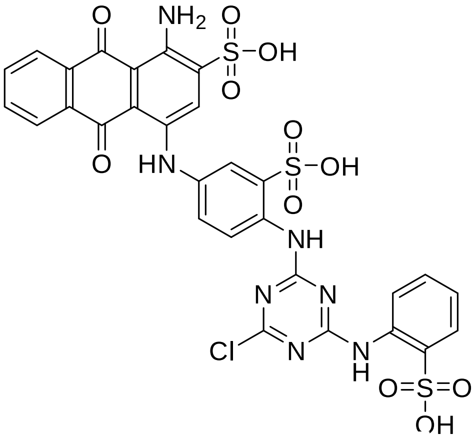

Cibacron Blue 3GA

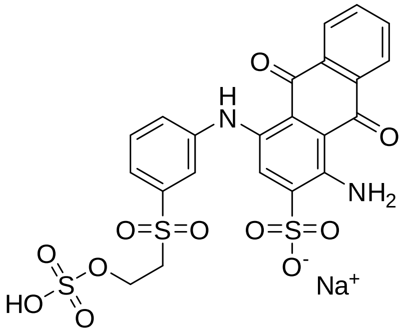

Reactive Blue 19

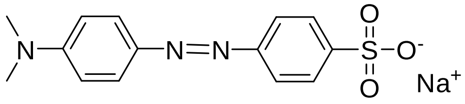

Methyl Orange

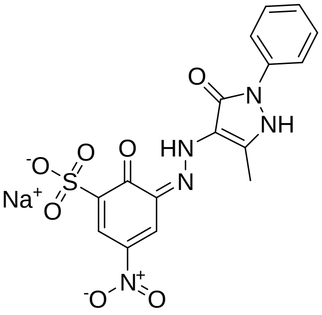

Acid Orange 74

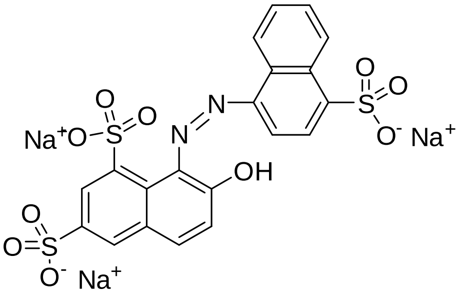

New Coccine

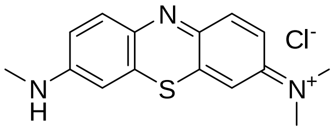

Azure B

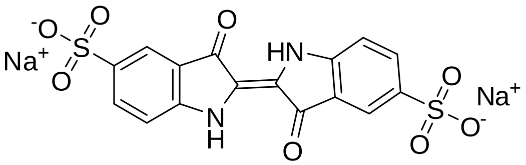

Indigo Carmine

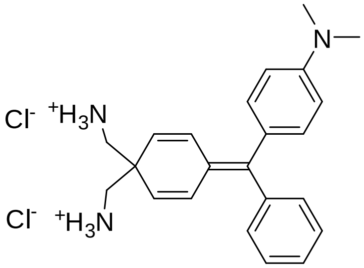

Malachite Green

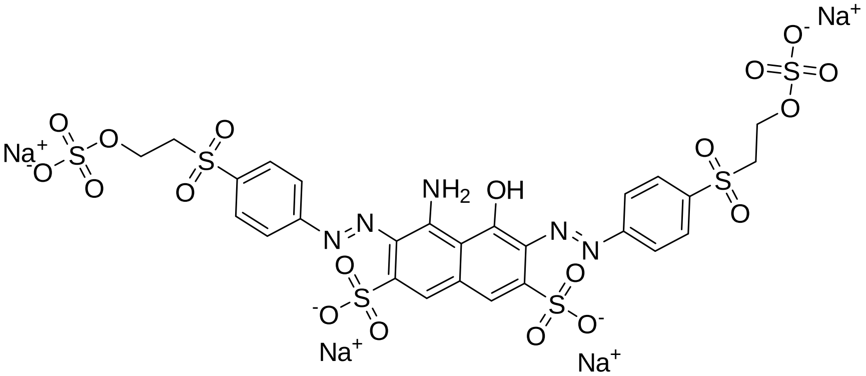

Reactive Black 5

Fig. S2. Structures of dyes used in this work. Pictures were generated with the ChemOffice 10.0 utilities (CambridgeSoft, U.K.).

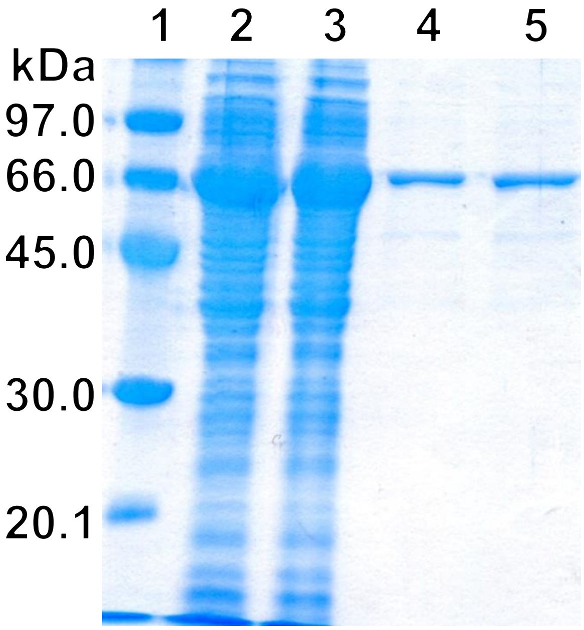

Fig. S3. SDS-PAGE analysis of BioF-CueO expression and purification. Lane 1, molecular weight markers; lane 2, total extract of IPTG-induced *E. coli* BL21 (DE3) [pDB2]; lane 3, soluble fraction from the extract of IPTG-induced *E. coli* BL21 (DE3) [pDB2]; lane 4, purified BioF-CueO by hydrophobic chromatography; lane 5, loading of PHA beads containing immobilized BioF-CueO protein.

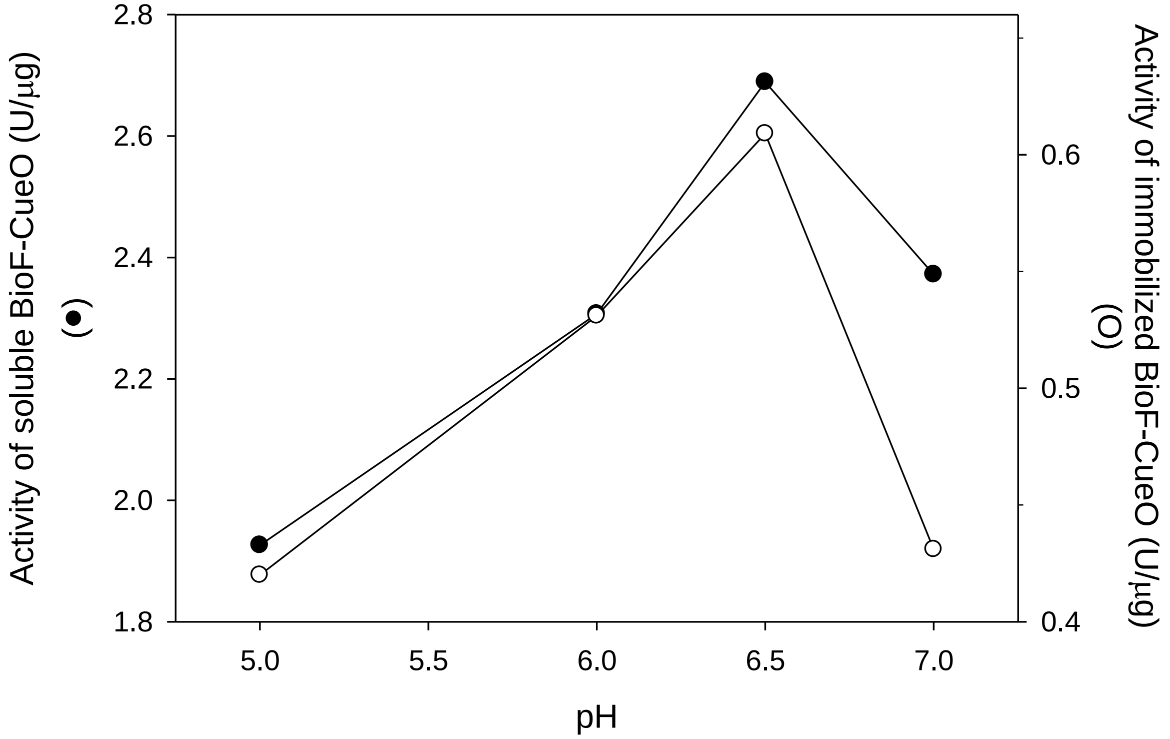

Fig. S4. pH dependence of BioF-CueO activity at 30 ºC both in aqueous solution and immobilized on PHB. 2,6-DMP (2 mM) was used as substrate in the presence of 10 µM Cu^{2+}. Both soluble and immobilized proteins were assayed at a final concentration of 1 µg/ml. Buffers used were 20 mM sodium acetate (pH 5.0) and Tris (pH 6.0-7.0).

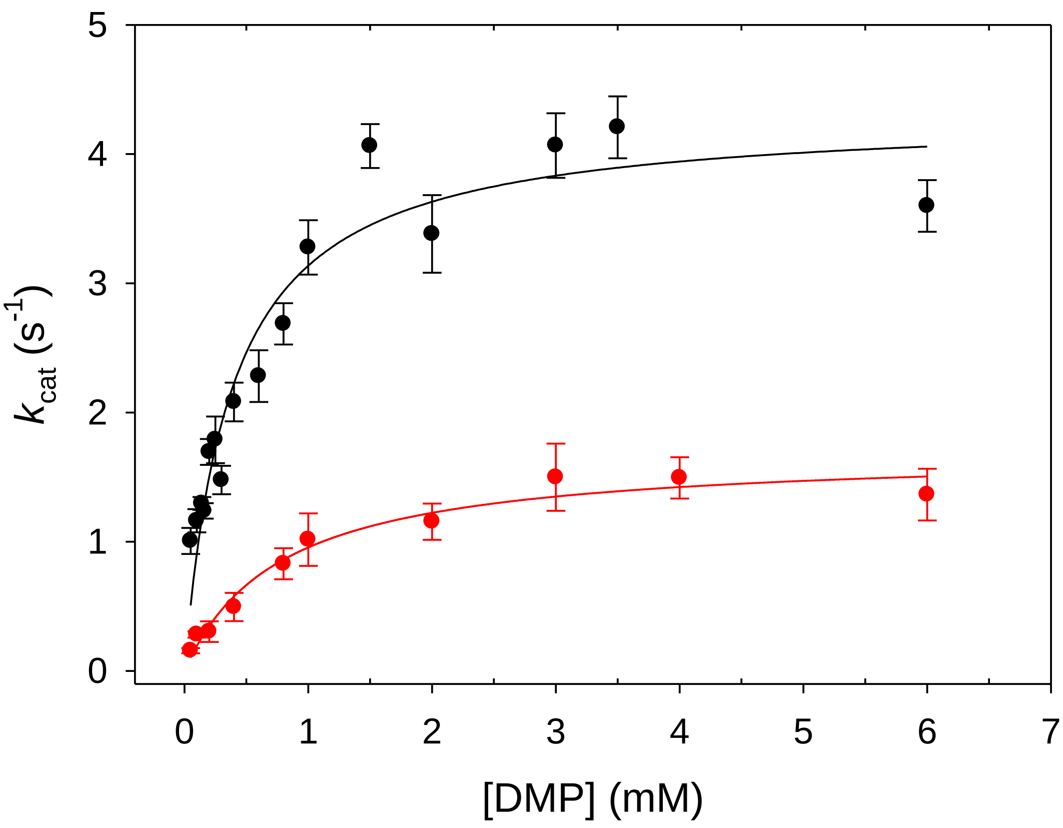

Fig. S5. Kinetic analysis of the activity of BioF-CueO in solution (black) and immobilized on PHB (red). Results are the mean of duplicates. See Table 1 for details.

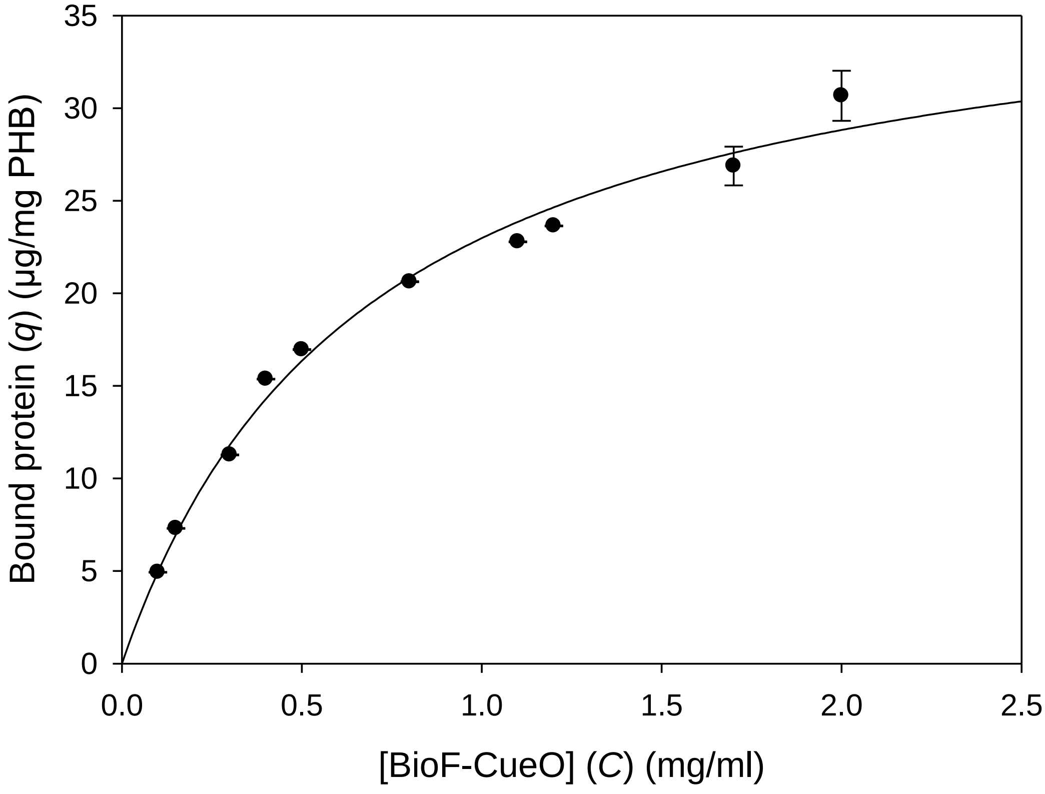

Fig. S6. Langmuir isotherm of BioF-CueO binding to PHB. Results are the mean of duplicates.

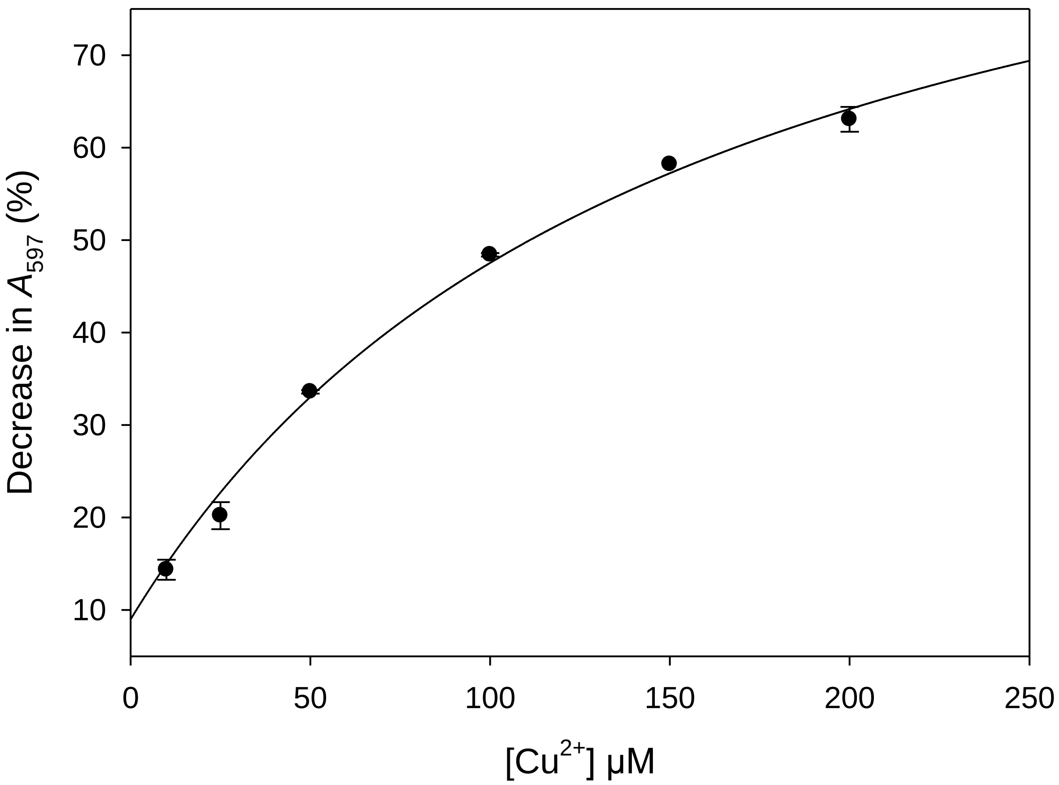

Fig. S7. Effect of Cu^{2+} concentration on the activity of BioF-CueO immobilized on PHB. One-ml solutions of RB5 were incubated in batch at 25 ºC for 1 h with PHB functionalized with BioF-CueO in the presence of varying Cu^{2+} concentrations. Results are the mean of triplicates.

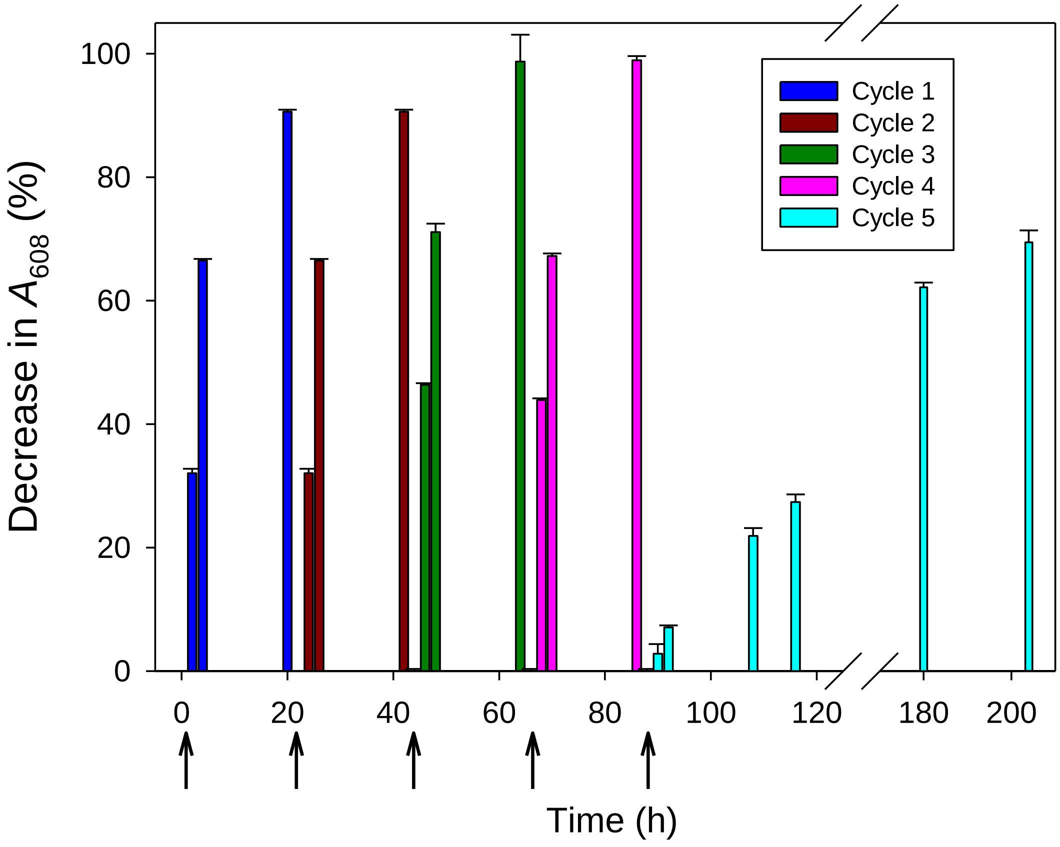

Fig. S8. Batch reutilization of PHB-immobilized BioF-CueO for decolorisation of Indigo Carmine (IC) solutions. At the times indicated by the arrows, fresh solutions of IC (20 ml) were mixed with PHB functionalized with BioF-CueO. Samples were removed at selected times to measure their absorbance at 608 nm. After a *c.a*. 20-hour incubation the whole solution was removed, the support was washed with buffer and then reloaded with a fresh dye solution. The last incubation cycle was expanded to 205 h due to the loss of enzymatic activity.

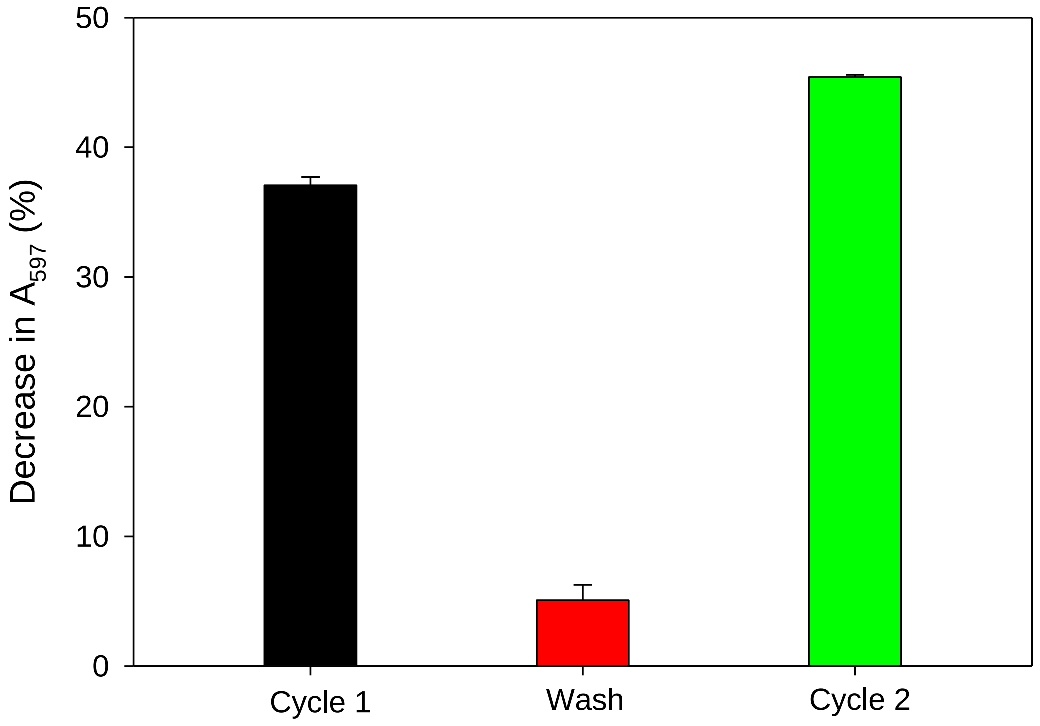

Fig. S9. Recycling of bioactive PHB support. A RB5 solution was incubated in batch in 20 mM Tris buffer, pH 7 plus 0.1 mM $CuSO_4$ at 25 ºC for 1 h with immobilized BioF-CueO. Then, the solution was removed (cycle 1) and the resin was washed with 2% (w/v) SDS for 15 min. After the detergent removal by extensive washing with buffer, the remaining activity was measured (wash). Finally, fresh protein was adsorbed on the PHB and its activity checked again for dye decolorisation (cycle 2). Results (mean of triplicates) are shown as decrease in absorbance with respect to the initial values.

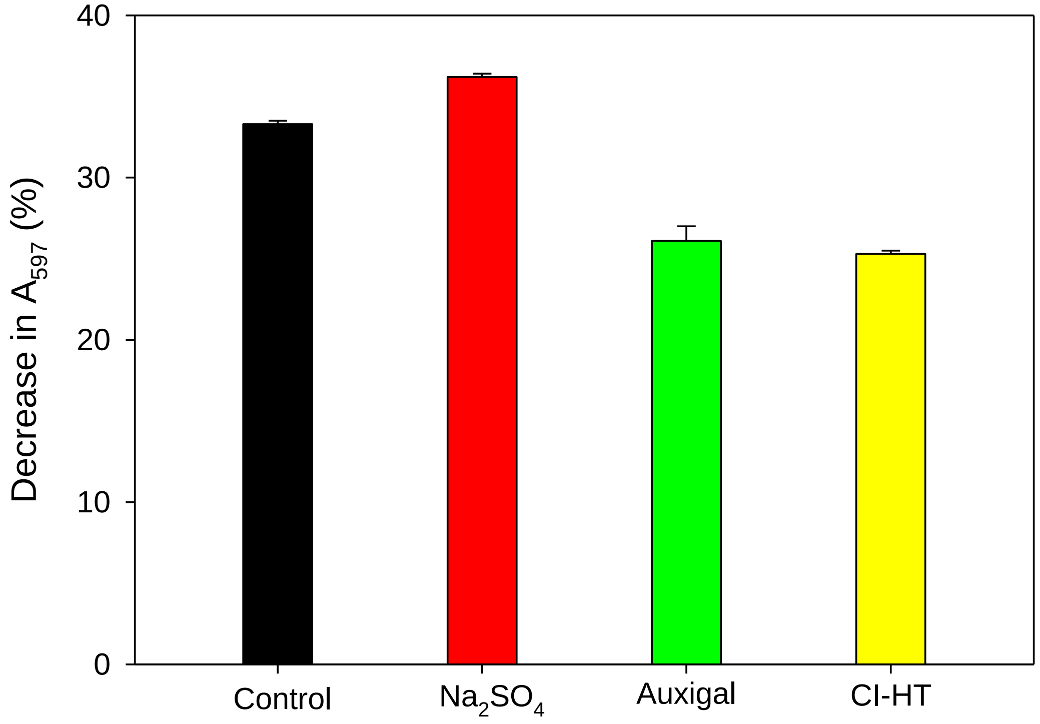

Fig. S10. Decolorisation of a RB5 solution in the presence of additives. An RB5 solution (1 mL) was incubated in batch on a digital rotary mixer (OVAN) for 1 h with PHB (around 0.1 mL bed volume) functionalized with BioF-CueO as described in Experimental Procedures. Results are the mean of triplicate experiments and are shown as the decrease in absorbance with respect to the initial values.
